# Challenges of Orthopaedic Practice During COVID-19 Outbreak: Multidisciplinary Collaboration is Required

**DOI:** 10.5704/MOJ.2007.026

**Published:** 2020-07

**Authors:** B Joob, V Wiwanitkit

**Affiliations:** 1 Private Academic Consultant, Bangkok, Thailand; 2 Department of Community Medicine, Dr D.Y. Patil Patil University, Pune, India

Dear Editor,

We would like to share ideas on the article “COVID-19 in Singapore and Malaysia: Rising to the Challenges of Orthopaedic Practice in an Evolving Pandemic^1^”.Tay et al. noted that *“Various medical specialties should utilise all available channels of communication to share our experience, learn from each other, and join force with our partners from other parts of the world to overcome this challenge^1^.”* Tay et al. presented several aspects in orthopedic surgery that are related to COVID-191. Practitioner has a chance to get contact with COVID-19 since the COVID patients might be asymptomatic and might enter into the orthopedic department of any medical center^2^. It is no doubt that collaboration is the key factor for successful containment of COVID-19 outbreak as mentioned by Tay *et al.* We should extend our collaboration beyond the scope of medicine. We should pay respect and work with other occupations for counteracting COVID-19. A simple example is the role of interior designer and engineer in reformatting of workplace to fit for the disease control during COVID-19 outbreak.
